# Neuropathy With Demyelinating Features in a Patient With Biallelic *HARS1* Variants

**DOI:** 10.1111/jns.70162

**Published:** 2026-08-13

**Authors:** Christina Del Greco, Allison R. Cale, Karl Haeberlein, Anthony Antonellis, Michael E. Shy, Tiffany Grider

**Affiliations:** ^1^ Department of Human Genetics University of Michigan Medical School Ann Arbor Michigan USA; ^2^ Department of Neurology University of Michigan Medical School Ann Arbor Michigan USA; ^3^ Department of Neurology, Carver College of Medicine University of Iowa Iowa City Iowa USA

**Keywords:** demyelinating neuropathy, *HARS1*, histidyl‐tRNA synthetase, loss‐of‐function effects, recessive disease

## Abstract

**Background and Aims:**

The *HARS1* gene encodes cytoplasmic histidyl‐tRNA synthetase, which catalyzes the ligation of histidine to tRNA^HIS^ in the cytoplasm as an early step in protein biosynthesis and is essential for cell viability. Pathogenic variants in *HARS1* have been associated with three phenotypes: autosomal dominant Charcot–Marie–Tooth (CMT) disease, a multisystem recessive syndrome with prominent ataxia, and autosomal recessive Usher syndrome. Here, we present a patient who is compound heterozygous for *HARS1* variants and who has a complex recessive phenotype that includes a neuropathy with demyelinating features and active denervation. Our computational and functional analyses support the pathogenicity of these alleles, suggesting that our findings expand the allelic and clinical heterogeneity of *HARS1*‐related disease.

**Methods:**

The individual found to have pathogenic compound heterozygous *HARS1* variants was evaluated in a neuromuscular clinic and was further investigated in research studies. Functional consequences of the *HARS1* variants were tested in yeast complementation assays; the phase of these alleles was confirmed via long‐range PCR and long‐read sequencing on DNA isolated from the proband, the mother, and the father.

**Results:**

A 25‐year‐old male with CMT1 and mild intellectual disability had a maternally inherited variant (p.Q410*) and a de novo variant in the *HARS1* gene (p.R375C) identified via trio exome sequencing. Studies in yeast revealed ablated function for p.Q410* and reduced function for p.R375C. Of 2480 informative sequencing reads generated from the proband: (a) 1765 (71%) included only p.R375C or only p.Q410*; (b) 237 (10%) included both alleles; and (c) 478 (19%) included neither allele.

**Interpretation:**

Studies in yeast revealed loss‐of‐function characteristics for both p.Q410* and p.R375C *HARS1*, consistent with these variants being pathogenic. Allele‐specific sequencing analyses are consistent with the proband having a compound heterozygous genotype and with p.R375C being a de novo variant that arose on the chromosome 5 transmitted by the father. There is therefore moderate evidence that the two identified *HARS1* variants are responsible for the recessive phenotype. This case report expands the allelic and phenotypic heterogeneity of biallelic *HARS1* pathogenic variants.

## 1 Background and Aims

Aminoacyl‐tRNA synthetases (ARSs) are an essential and ubiquitously expressed family of enzymes responsible for ligating tRNA molecules with cognate amino acids in the cytoplasm and mitochondria of human cells [1]. The human nuclear genome encodes 37 ARS loci: 18 encode enzymes that exclusively charge tRNAs in the cytoplasm; 17 encode enzymes that exclusively charge tRNAs in the mitochondria; and two encode enzymes that charge tRNAs in both cellular compartments via a variably employed mitochondrial targeting sequence. Gene nomenclature for the ARS loci leverages the single‐letter code for amino acid in question, the acronym “ARS,” and a “1” or a “2” if charging takes place in the cytoplasm or mitochondria, respectively (e.g., the cytoplasmic enzyme responsible for charging tRNA molecules with histidine is encoded by *HARS1*).

Over the past two decades, all 37 ARS loci have been implicated in a range of inherited human diseases. First, all 37 have been implicated in myriad recessive phenotypes with frequent involvement of the central nervous system including developmental delay, intellectual disability, microcephaly, and epileptic encephalopathy. In addition, tissue predominant phenotypes have been observed, which can vary depending on the ARS gene that is mutated. For example: biallelic mutations in lysyl‐tRNA synthetase (*KARS1*) have been implicated in sensorineural hearing loss; biallelic mutations in leucyl‐tRNA synthetase (*LARS1*) have been implicated in infantile hepatopathy; and biallelic variants in cysteinyl‐tRNA synthetase (*CARS1*) have been implicated in brittle hair and nails [1]. Second, seven ARS loci (*AARS1*, *GARS1*, *HARS1*, *NARS1*, *SARS1*, *WARS1*, and *YARS1*) have been implicated in dominantly inherited peripheral neuropathy [1]. Interestingly, both recessive and dominant ARS‐related disease are associated with peripheral neuropathy, suggesting that there are shared mechanistic features.

As noted above, *HARS1* encodes the enzyme responsible for charging tRNA with histidine in the cytoplasm (*HARS2* encodes the mitochondrial enzyme). Pathogenic variants in *HARS1* have been associated with three distinct phenotypes: (1) homozygosity for the p.Y454S *HARS1* variant has been associated with Usher Syndrome Type III, which is characterized by hearing loss and retinitis pigmentosa [2]; (2) compound heterozygosity for loss‐of‐function *HARS1* variants has been associated with a multisystem syndrome with prominent ataxia [3]; and (3) heterozygosity for loss‐of‐function *HARS1* missense variants has been associated with Charcot–Marie–Tooth (CMT) Disease Type 2W, which is characterized by a motor and sensory axonal neuropathy [4]. Here, we present a patient who is compound heterozygous for *HARS1* variants and who has a complex recessive phenotype that includes a neuropathy with demyelinating features and active denervation. Our computational and functional analyses support the pathogenicity of these alleles, suggesting that our findings expand the allelic and clinical heterogeneity of *HARS1*‐related disease.

## 2 Case Report

**FIGURE 1:**
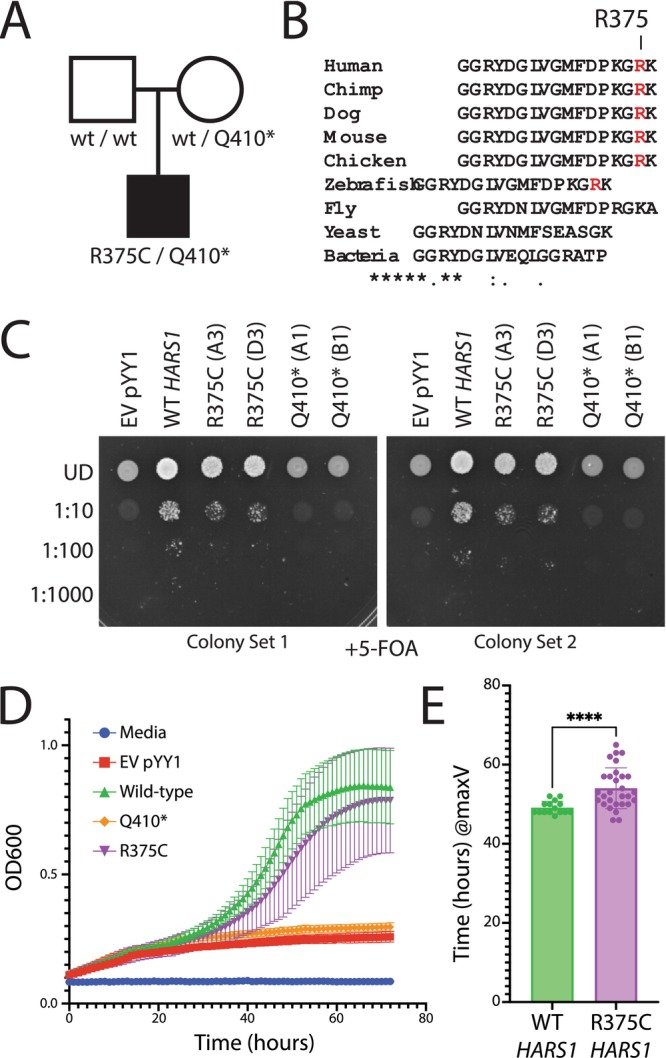
Loss‐of‐function *HARS1* alleles in a patient with recessive disease. (A) Pedigree depicting inheritance of the p.R375C and p.Q410* *HARS1* alleles. Squares represent males, circles represent females, and filled shapes indicate affected individuals. Genotypes are indicated below (“wt” indicates a wild‐type allele). (B) Multiple‐species alignment depicting the conservation of arginine 375 (indicated in red text) and the surrounding amino‐acid residues. Species are indicated on the left and conservation is assessed along the bottom with “*” indicating identical amino acids at that position, “:” indicating only conservative substitutions at that position, and “.” indication only semiconservative substitutions at that position. (C) Yeast complementation assays using a humanized model. Each yeast strain was transformed with a *LEU2*‐bearing vector encoding wild‐type human *HARS1* (WT), p.R375C *HARS1*, p.Q410* *HARS1*, or no insert (“EV pYY1”). Cultures for each strain (labeled along the top) were spotted on medium containing 5‐FOA (undiluted or diluted; dilutions are indicated on the left) to determine if the human *HARS1* alleles complement loss of endogenous *HTS1*. Two independently derived expression constructs were developed to study p.R375C (A3 and D3) and p.Q410* (A1 and B1), and two colonies from each transformation (colony sets 1 and 2) were studied in complementation assays. (D) Growth curve and (E) *V*_max_ analysis showing that p.R375C grows more slowly compared to wild‐type *HARS1*, consistent with a hypomorphic allele. ****p.(0,0001).

This study was approved by—and subjects enrolled through—the University of Iowa Institutional Review Board (IRB # 201201787). The proband (Figure 1A; filled in square) is a 24‐year‐old male with moderate intellectual disability, congenital weakness, progressive bilateral cavovarus, upper and lower extremity distal muscle weakness, length‐dependent sensation loss, areflexia, scoliosis, and a steppage gait. He was the product of a full‐term spontaneous vaginal birth, remarkable for meconium staining but otherwise uncomplicated. Attainment of developmental milestones for both motor and language functions was delayed. He did not walk independently or use single words until 2.5–3 years of age. He was never able to run. He received speech/language, occupational, and physical therapies starting at the age of 18 months and these services continued with his public school district. He had a diagnosis of congenital velopharyngeal insufficiency with surgical management by a wide pharyngeal flap at age 7. Diagnosis of CMT type 1 was made at age 10 with neurological evaluation including nerve conduction (Table 1). There is no family history of CMT disease or intellectual disability. The mother of the proband was briefly examined by a neurologist, which revealed normal intellect, normal foot structure, normal reflexes, and normal gait; nerve conduction studies were not performed on the mother. The proband's brain and spine MRI were normal. Hearing and vision screening were normal. Neuropsychological testing was consistent with moderate intellectual disability.

**TABLE 1:** Nerve conduction study results.

Motor nerve conduction studies			
Nerve	Latency (ms) (normal)	CMAP amplitude (mV) (normal)	NCV (m/s) (normal)
Left median	Wrist 5.5 (4.2) Elbow 13.6 (8.8)	Wrist 5.9 (3.5) Elbow 4.9 (3.5)	Elbow 29.0 (> 48)
left ulnar	Wrist 4.1 (3.4) Below elbow 10.1 (7.5) Above elbow 13.2 (9.6)	Wrist 4.8 (2.8) Below elbow 4.2 (2.8) Above elbow 4.1 (2.8)	Wrist‐below elbow 38.3 (> 49) Below elbow‐above elbow 32.3 (> 50)
Right peroneal (EDB)	NR	NR	NR
Right tibial (AH)	NR	NR	NR

Sensory nerve conduction studies		
Nerve	SNAP amplitude (μV) (normal)	SCV (m/s) (normal)
Left median	NR	NR
Left ulnar	NR	NR
Left radial	NR	NR

Abbreviations: CMAP, compound muscle action potential; NCV, nerve conduction velocity; NR, no response; SCV, sensory conduction velocity; SNAP, sensory nerve action potential.

A chromosome microarray was normal. Very long chain fatty acids were also normal. After a negative demyelinating CMT panel, trio exome sequencing revealed several variants of interest. A maternally inherited hemizygous VUS in *DMD* (p.K20963M); however, the proband has normal CK levels. One heterozygous “likely pathogenic” in *COL6A3* (c.8966+1G>C) and one pathogenic variant (p.H1069Q) in *ATP7B* consistent with being an unaffected carrier of autosomal recessive myopathy and Wilson's disease, respectively; in support of this, copper and ceruloplasmin levels were normal. A heterozygous “likely pathogenic” maternally inherited variant (c.1228C>T; p.Q410*) and a second de novo variant (c.1123C>T; p.R375C) in the *HARS1* gene (NM_002109). Based on the clinical phenotype and inheritance pattern, we nominated the *HARS1* genotype as a possible explanation for the presumptive recessive phenotype in the proband.

A previous report on biallelic *HARS1* variants [3] describes three affected individuals from two families with a complex phenotype that included ataxia. In sum, these patients were described as having a shuffling, ataxic gait with leg spasticity, upper limb tremor, motor function delay, severe distal weakness in the lower limbs, *pes planus* with hammertoe, decreased motor nerve conduction velocities, intellectual disability, speech delay and/or dysarthric speech, and nystagmus. Thus, there is some phenotypic overlap between the proband reported here and previous reports of biallelic variants; however, the motor sensory neuropathy with demyelinating features is currently the predominant phenotype in the patient we report here, without obvious symptoms of cerebellar ataxia.

## 3 Interpretation

Segregation analysis of the two variants identified in the proband (p.Q410* and p.R375C) revealed that the proband's mother is heterozygous for p.Q410* and that neither parent carries p.R375C (Figure 1A). Interestingly, the p.R375 codon (CGC) contains two CpG dinucleotides (one in the forward and one in the reverse complemented orientation). If the cytosine of the CpG dinucleotide in the forward orientation were to be methylated and deaminated to a thymine, this would result in a codon for cysteine (TGC). This supports the observation that p.R375C was not seen in either parent and is a de novo variant in the proband. To determine if the p.R375C variant resides on the same allele as p.Q410* (i.e., resulting in the proband being heterozygous for a complex allele) or if it resides on the allele transmitted from the father (i.e., resulting in the proband being compound heterozygous for the two variants), we subjected DNA isolated from the proband, the mother, and the father to long‐range PCR and long‐read DNA sequencing analysis (Plasmidsaurus “premium PCR” amplicon sequencing); this was possible due to the two *HARS1* variants being separated by only ~2 kb on human chromosome 5. These analyses confirmed that the mother is heterozygous for p.Q410* and that the father does not carry either mutation. Of 2480 informative sequencing reads generated from the proband: (a) 1765 reads (71%) included only p.R375C or only p.Q410*; (b) 237 reads (10%) included both alleles; and (c) 478 reads (19%) included neither allele. These data are consistent with the proband having a compound heterozygous genotype and with p.R375C being a de novo variant that arose on the chromosome 5 transmitted by the father. Please note that the above data (i.e., the reads including both variants and the reads containing neither variant) are also consistent with a significant amount of PCR chimerism, an artifact in which an incomplete DNA extension product anneals to a template from the opposite allele in genomic DNA amplification. The DNA polymerase then extends this product, creating a recombinant amplicon containing sequences from two distinct alleles.

To further interrogate the potential pathogenicity of the two identified *HARS1* variants, we determined the frequency of each allele in the gnomAD variant database and assessed the conservation of the p.R375 codon via multispecies protein sequence alignments using CLUSTAL W; since p.Q410* results in a premature stop codon that would be predicted to delete part of the catalytic domain and all of the tRNA recognition domain [4], assessing the conservation of p.Q410 would not provide valuable information. We found that p.Q410* is absent from the gnomAD variant database and that p.R375C is detected on 52 out of 1 614 074 alleles with no homozygous individuals. These data are consistent with the population frequency of other ARS alleles implicated in recessive disease. Finally, conservation assessments revealed that p.R375 resides at a residue conserved among vertebrate species (Figure 1B), indicating that this residue may be important for gene function.

To determine the functional consequences of p.R375C and p.Q410* *HARS1*, we tested them in a previously described humanized yeast complementation assay [3]. Here, the endogenous yeast gene *HTS1* has been deleted (Horizon Discovery) and viability is maintained via a centromere‐bearing construct that expresses both wild‐type *HTS1* and the auxotrophic marker *URA3*. We cloned the wild‐type or mutant human *HARS1* open reading frame into a second vector (YY1, a high‐copy vector harboring a *LEU2* auxotrophic marker) and transformed the above yeast strain with either an “empty” YY1 construct with no *HARS1* insert or a YY1 construct to express either wild‐type or mutant *HARS1*. Upon growing the strain on media containing 5‐FOA, which converts the *URA3* gene product into a lethal compound and selects for cells that have spontaneously lost the *URA3*‐bearing maintenance vector, viability is now dependent on the function of histidyl‐tRNA synthetase encoded on the YY1 construct. First, transforming the yeast strain with an empty vector (“EV” in Figure 1C) resulted in no yeast cell growth, consistent with *HTS1* being an essential gene. Second, transforming the yeast strain with a vector to express wild‐type human *HARS1* resulted in yeast cell growth (Figure 1C), indicating that the human gene can at least partially rescue deletion of the yeast ortholog *HTS1*. Finally, transforming the yeast strain with a vector to express p.R375C *HARS1* resulted in visibly decreased cell growth (Figure 1C) and transforming the yeast strain with a vector to express p.Q410* *HARS1* resulted in no cell growth (Figure 1C). These data are consistent with p.R375C *HARS1* being a hypomorphic allele and with p.Q410* *HARS1* being a functionally null allele. To quantitate the reduced yeast growth associated with p.R375C *HARS1*, we performed yeast growth curve analyses using a BioTek Epoch2 Microplate Spectrophotometer. This revealed a shift in the p.R375C growth curve to the right compared to wild‐type *HARS1* (Figure 1D), representing a 5‐h increase at the *V*_max_ time (Figure 1E; 49 h for wild‐type *HARS1* compared to 54 h for p.R375C *HARS1*). As before, the empty vector and the p.Q410* variant were associated with no yeast growth in this assay (Figure 1D,E). In summary, our computational and functional analyses support the pathogenicity of p.R375C and p.Q410* *HARS1* in the observed recessive phenotype. Based on current American College of Medical Genetics (ACMG) and Association for Molecular Pathology (AMP) guidelines, we conservatively conclude that there is “moderate” evidence for the pathogenicity of p.R375C and p.Q410* (Table 2) [5].

**TABLE 2:** Assessments of pathogenicity based on ACMG guidelines.

*HARS1* variant	Evidence of variant impact	New ACMG classification
c.1123C>T (p.R375C)	PM2 (mod), PS3 (mod), PM3 (mod)	Likely pathogenic
c.1228C>T (p.Q410*)	PM2 (mod), PVS1 (mod), PS3 (mod)	Likely pathogenic

*Note:* Evidence of variant impact descriptions: PM2: extremely low frequency in gnomAD population databases; PS3: well‐established functional studies show damaging effect on the gene or gene product; PM3: for recessive disorders, detected in trans with a pathogenic variant, or in a homozygous or compound heterozygous state in affected cases; PVS1: Null variant in a gene where loss of function is a known mechanism of disease [5].

## Funding

Christina Del Greco and Allison R. Cale are supported by the Michigan Pre‐Doctoral Training Program in Genetics (GM149391). Christina Del Greco is also supported by an NIH National Research Service Award from the National Institute for Child Health and Human Development (F31HD111131). Anthony Antonellis is supported by a grant from the National Institute of General Medical Sciences (GM136441). These studies were also funded, in part, by a grant from the Charcot–Marie–Tooth Disease Associate to the Inherited Neuropathy Consortium.

## Conflicts of Interest

The authors declare no conflicts of interest.

## Data Availability

No large datasets were generated in the course of this study. Other data that support the findings of this study are available on request from the corresponding author. The data are not publicly available due to privacy or ethical restrictions.
